# Low power, less occupying area, and improved speed of a 4-bit router/rerouter circuit for low-density parity-check (LDPC) decoders

**DOI:** 10.12688/f1000research.73404.1

**Published:** 2022-01-05

**Authors:** Chinnaiyan Senthilpari, Rosalind Deena, Lee Lini

**Affiliations:** 1 Faculty of Engineering, Multimedia University, Cyberjaya, Selangor, 63100, Malaysia

**Keywords:** LDPC decoder, multiplexer, demultiplexer, pass transistor logic

## Abstract

**Background:** Low-density parity-check (LDPC) codes are more error-resistant than other forward error-correcting codes. Existing circuits give high power dissipation, less speed, and more occupying area. This work aimed to propose a better design and performance circuit, even in the presence of noise in the channel.

**Methods:** In this research, the design of the multiplexer and demultiplexer were achieved using pass transistor logic. The target parameters were low power dissipation, improved throughput, and more negligible delay with a minimum area. One of the essential connecting circuits in a decoShder architecture is a multiplexer (MUX) and a demultiplexer (DEMUX) circuit. The design of the MUX and DEMUX contributes significantly to the performance of the decoder. The aim of this paper was the design of a 4 × 1 MUX to route the data bits received from the bit update blocks to the parallel adder circuits and a 1 × 4 DEMUX to receive the input bits from the parallel adder and distribute the output to the bit update blocks in a layered architecture LDPC decoder. The design uses pass transistor logic and achieves the reduction of the number of transistors used. The proposed circuit was designed using the Mentor Graphics CAD tool for 180 nm technology.

**Results:** The parameters of power dissipation, area, and delay were considered crucial parameters for a low power decoder. The circuits were simulated using computer-aided design (CAD) tools, and the results depicted a significantly low power dissipation of 7.06 nW and 5.16 nW for the multiplexer and demultiplexer, respectively. The delay was found to be 100.5 ns (MUX) and 80 ns (DEMUX).

**Conclusion:** This decoder’s potential use may be in low-power communication circuits such as handheld devices and Internet of Things (IoT) circuits.

## Introduction

Low-density parity-check (LDPC) codes are considered more error resistant when compared to other forward error-correcting codes. These error-based circuits have been proved by their performance in the presence of noise in the channel. ^1^ Hence, LDPC decoders have been used more actively for communication applications. Different approaches may be used in the design of an LDPC decoder. One such structure is the layered approach, consisting of a layered design, memory unit, computational block, full adders, parity check unit, bit update unit, and router/reverse router circuits. ^2^ The decoding process begins with data being received into the decoder through the bit update block. The bit update block receives data, arranges them into their vectors according to the system requirements, and stores them. These data are routed to the parallel adder through the routing circuit and the data bus. The parallel adder now computes the memory block stored in the previous iteration and the new vector. The output of the computation is checked for errors using the parity checker. ^3^ The result goes through another computation process to generate the original vector stored in the bit update unit for the next iteration. Also, new values after the parity check are stored in the memory block.

Routers form an integral part of this architecture, sending data bits through the routers’ different layers. Routers are multiplexer or demultiplexer circuits used to select appropriate data to be sent or to distribute the received data bits to other units. Multiplexers (MUX) and Demultiplexers (DEMUX) form the basic units of data paths. They are used in applications like processor buses in CPUs, network switches, and digital signal processing stages, involving resource sharing and graphic controllers. In large-scale systems, multiplexers aid in the reduction of integrated circuits used in some designs. In this research, the design of the multiplexer and demultiplexer is achieved using pass transistor logic. ^4^ According to existing authors of the multiplexer, demultiplexer, and LDPC encoder circuits, a higher number of transistors leads the critical path and results in higher power dissipation. ^5^

The proposed method reduced the number of transistors in the design and the regular arrangement of transistors, thereby reducing the critical path. The target was low power dissipation, improved throughput, and smaller delay with a minimum area. Low power design is essential when this circuit is used along with many other components for communication purposes. Pass Transistor Logic (PTL) has the advantage of reducing the number of transistors by eliminating redundant transistors. Here the transistors act as switches to pass different logic levels between nodes of a circuit. This paper’s main objective was to design and develop routers and bit update blocks for the LDPC decoder. The proper design of the router, rerouted, and LDPC circuit reduces the critical path, power dissipation, and speed increases. This paper reviews the related work in designing multiplexers and demultiplexers and describes the design methodology used in the proposed circuits. The results obtained from the simulation are analyzed, and conclusions are then made regarding the proposed circuits.

### Literature review

Unlike the main building blocks such as the adders and parity checkers, routers form a crucial support system to the decoder. The routers’ function, mainly comprised of multiplexers and demultiplexers, helps arrange data bits according to the system configuration and passes the information through appropriate layers. Binary signals control multiplexers. ^2^ The analogue MUX/DEMUX was designed using ternary inverters to control the circuits, and CMOS transmission gates were used. ^6^^–^^8^ The design improved and proved to be excellent for ternary inverters. With the idea of switching activities suggested by Anitha and Javachitra, ^9^ adiabatic logic reduces the power by offering back the stored energy to the supply and this was used for the 16:1 multiplexer and 1:16 demultiplexer. The results indicated that they had less power dissipation than conventional CMOS circuits. An 11 Gb/s CMOS demultiplexer using redundant multi-valued logic (RMVL) was proposed by Ahn and Kim (2006). ^10^ The circuit received serial binary data, which was converted to parallel redundant multi-valued data. The converted data are reconverted to parallel binary data. This makes it possible to achieve higher operating speeds than that of conventional binary logic. The implemented DEMUX consisted of eight integrators and was designed with a 0.35 μm standard CMOS process. The DEMUX achieved the maximum data rate of 11 Gb/s and the average power consumption of 69.43 mW. This circuit was expected to operate faster than 11Gb/s in the high operating frequency’s deep-submicron process ***.*** A demultiplexer has been designed with 36 transistors using 90 nm CMOS technology. ^7^ Auto-generation technique and semi-custom layout design were integrated. There was an improvement in power consumption and area due to the semi-customized demultiplexer layout.

## Methods

The router circuit in a decoder is a bank of MUX and DEMUX that forward the appropriate estimate terms from memory to the corresponding bit update circuit. The proposed MUX, DEMUX, bit update circuit, and proposed LDPC circuits logic simulations are executed mainly to validate the circuit’s functionality. The designed circuit had the required logic behaviour. In the layout, the memory cell’s charging and discharging were validated by the aspect ratio factor and expressed with current scaling methods. The proposed circuits were validated by reliable, optimum data of the designed parameters. Modern communication systems demand high reliability and optimum data rate, which makes the standards for future communication technology move towards methods of error correction that enable high throughput decoding with optimum performance based on the Shannon capacity.

### Multiplexer (MUX)

**Figure 1.**
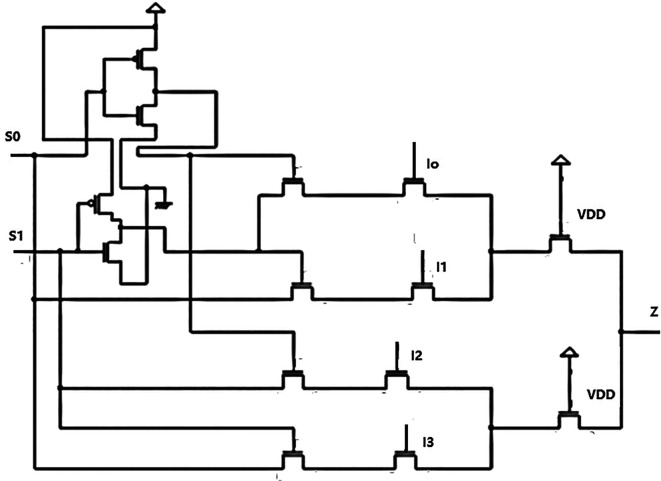
Proposed circuit of the 4 × 1 multiplexer.

The multiplexer is a combinational logic circuit that selects an appropriate analogue (or) digital signal from several input signals and forwards it to a single output line. ^11^ A multiplexer has several input lines and a single output line. The selection of the appropriate input is based on unique control lines called select lines. Figure 1 depicts a basic multiplexer with four inputs I _0_, I _1_, I _2_, I _3,_ and a single output line (Z). Multiplexers can be designed for a 2 ^n^ number of inputs. In this design, we used a 4 × 1 MUX because it is simpler to cascade these circuits for many inputs, and the decoder was also for 4-bit data. There are two select lines, S _0_, S _1_ which are the control lines of the circuit. The MUX is 4 × 1, representing four inputs and one output. An additional set of input lines control each input line’s selection according to these control input’s binary conditions, which indicated ‘HIGH’ (1) or ‘LOW’ (0). Multiplexers have an even number of 2 ^n^ data input lines and some control inputs that match the number of data inputs. The truth table for the 4 × 1 MUX is shown in Table 1.

**Table 1.** Truth table for the proposed 4 × 1 MUX.

Select inputs		Inputs				Output
S1	S0	I _0_	I _1_	I _2_	I _3_	Z
0	0	**I**_**0**_	×	×	×	I _0_
0	1	×	**I**_**1**_	×	×	I _1_
1	0	×	×	**I**_**2**_	×	I _2_
1	1	×	×	×	**I**_**3**_	I _3_

The output Z is obtained from the Boolean expansion.

$$Z=I_{0}\bar{S_{1}}\;\bar{S_{0}}+I_{1}\bar{S_{1}}S_{0}+I_{2}S_{1}\bar{S_{0}}+I_{3}S_{1}S_{0}.$$ (1)

The equation (1) was expanded using associative and commutative laws to obtain an appropriate and optimized circuit equation for implementing the multiplexer. ^11^ Any single input line is selected instantly depending on the combination of select lines input to be connected to the output Z. For example, if the select lines combination S _1_S _0_ is a logic ‘0’ a logic ‘1’ (01), then input line I _1_ would be connected to the output Z, referring to Table 1. Adding more control address lines (n) allowed the multiplexer to control more inputs to switch 2 ^n^ inputs. Still, each control line configuration will connect only one input to the output. In our proposed circuit, optimization of the circuit is done using pass transistor logic to design the multiplexer.

A 4 × 1 MUX was designed as shown in Figure 1, and the input to the multiplexer in this circuit was from a bit update block (BUB), part of the LDPC decoder structure. The inputs were from the 4-bit update units used in the decoder circuit designed for this research. The multiplexer aimed to receive the updated data bits from the bit update unit and rearrange the vectors according to the circuit’s requirements. ^12^ The multiplexer circuit was designed using pass transistor logic. The MUX comprised NMOS and PMOS circuits for the inverters and only NMOS circuits for the remaining circuit. The inverter complemented the select input signals S _0_(S _A_) and S _1_(S _B_). The multiplexer was configured to have series-connected switches so that, based on the input combination of S _0_ and S _1_, one of the inputs was selected to pass the input to the output. The multiplexer passed a signal when the controlling voltage was logic low.

The circuit used NMOS because electron mobility is better than hole mobility, and hence the performance will be better. The inputs I _0_, I _1_, I _2_, and I _3_ fed from the 4-bit update circuits had the bit update unit’s computation values. The selection of the input that was given to the router was based on the select inputs S _1_ and S _0_. Inputs I _0_, I _1_, I _2_, and I _3_ were chosen to connect to the output line Z. Assuming the select inputs had an input combination of S _0_ = 0 and S _1_ = 1. The S _0_ input was fed to an inverter circuit formed by the pass transistors, which passed the value ‘0’ to the circuit, and the S _1_ with a logic ‘1’ was given to the other inverter circuit. The NMOS controlled the ground and the output in one inverter circuit, while PMOS connected the input supply V _DD_ and the output. ^13^ The transistors then did what they are best designed for, that is the NMOS allowed a logic ‘0’, and the PMOS allowed a logic ‘1’. It acted like a 2 × 1 MUX, where the inputs are logic 0 and logic 1. The input variable acted as the control signal and determined which input should be sent to the output. Hence, a combination of both inverters at the input would help select the signal sent to the output. This would be either I _0_, I _1_, I _2_, or I _3._ In our example, I _2_ was fed to the output Z = I _2_.

Multiplexer design can be enlarged to have many more inputs by using the basic multiplexer circuits. A 16 × 1 MUX can be designed using 2 × 1, 4 × 1, and 8 × 1 MUX. As per basic MUX circuit design, 4 × 1 multiplexers are used so 16 inputs are available. Inputs I _0_ to I _3_ (for bits zero to three) are for the first multiplexer (to PMOS), I _4_ to I _7_ (for bits four to seven) to the second, and so on where the last multiplexer has inputs I _12_ to I _15_ (for bits 12 to 15). Every multiplexer’s select inputs are combined in parallel into two main selection lines that connect all four multiplexers. ^14^^,^^15^ The output from each multiplexer is now fed as four inputs to another 4 × 1 multiplexer. The output from this multiplexer becomes the main output of the circuit.

### Demultiplexer (DEMUX)

A demultiplexer is a combinational circuit that routes a single input line to multiple digital output lines. The demultiplexer of 2 ^n^ outputs has ‘n’ select lines to select which output lines need to be connected to the input. ^13^^,^^14^ In simple terms, it is a data distributor. The demultiplexer is a 1 × 4 unit, implying a single input line Y and four output lines, D _0_, D _1_, D _2,_ and D _3_. There are two select lines, S _0_ and S _1_. The select lines help to decide to which output line the input line Y should be connected. The select lines are controlled by the binary combination of 0 and 1. The select lines S _0_ and S _1_ can take on 00, 01, 10, and 11. These are the four possible combinations for two input signals and hence four possible output lines. The combination and connection of input Y to the output lines D _0_, D _1_, D _2,_ and D _3,_ follow the truth table given in Table 2. The data input to be connected to the particular output line is obtained from the equation,

$$Y={\bar{S}}_{1}{\bar{S}}_{0}D_{0}+\bar{S_{1}}S_{0}D_{1}+S_{1}\bar{S_{0}}D_{2}+S_{1}S_{0}D_{3}.$$ (2)

**Table 2.** Truth table for the proposed 1 × 4 demultiplexers.

Data input	Select inputs		Outputs			
Y	S _1_	S _0_	D _0_	D _1_	D _2_	D _3_
Y	0	0	1	×	×	×
Y	0	1	×	1	×	×
Y	1	0	×	×	1	×
Y	1	1	×	×	×	1

**Figure 2.**
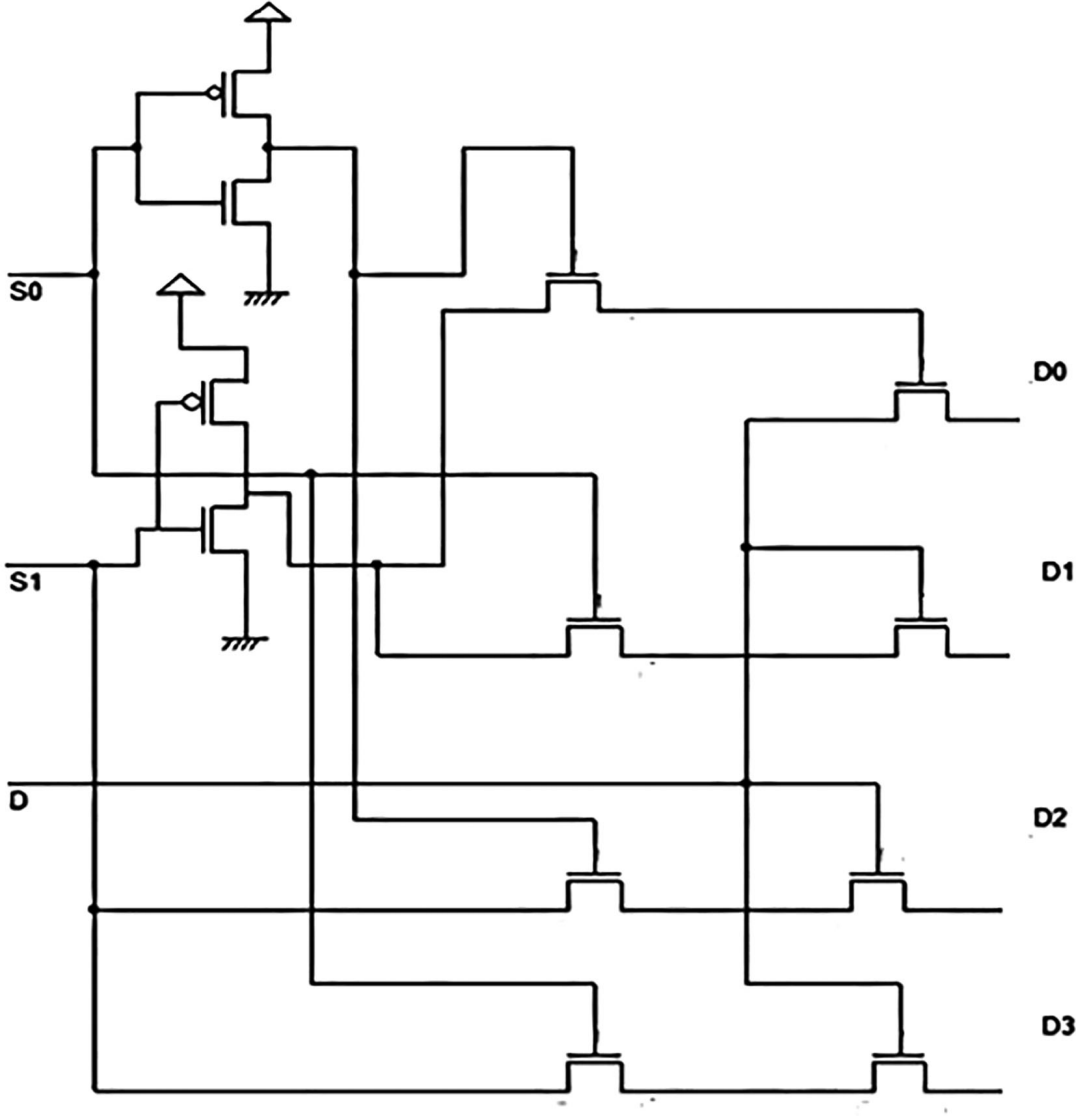
Proposed circuit of the 1 × 4 demultiplexers.

The Boolean expression in equation (2) is obtained from the truth table in Table 2, which can be implemented using basic logic gates or CMOS logic. Adding more address line inputs, it is possible to switch more outputs giving 1-to-2 ^n^ data line outputs. ^16^ The proposed demultiplexer was also a 1 × 4 demultiplexer constructed using pass transistor logic, as shown in Figure 2. In the figure, two inverter circuits form the input point for the DEMUX. The inverters were constructed with opposite polarity Metal Oxide Semiconductor Field Effect Transistors (MOSFETs) with their gates connected to form the input voltage V _in_ shown as S _A_ and S _B_. The drain terminals of both MOSFETs were connected to form a typical output. ^17^ These MOSFETS were connected in such a way (complementary) that only one MOSFET conducts when the input has a low or high input voltage due to the complementary connection.

The Gate-Source voltage V _GS_ is equal to V _in_, that is:

$$V_{\mathrm{GS}}=V_{\text{in}}\ldots .\text{for NMOS}$$

and the Source-Gate voltage given by V _SG_ is:

$$V_{\mathrm{SG}}=V_{\mathrm{DD}}–V_{\text{in}}\ldots .\text{for PMOS}$$

Where V _DD_ is the supply voltage, the input voltage can have values from 0 to V _DD_. When S _A_ = V _in_ = V _DD_, the PMOS transistor gets cut off while the NMOS conducts and current flows to the ground terminal, and the output voltage is ‘0’. The ‘0’ volts are now applied to one of the inputs of transistor T5, which is in series with T6.

If input S _B_ had an input value of ‘0’ volts, the NMOS transistor inverter was cut off while PMOS conducted to give a path to the power supply and the output now had a value V _DD_. The second input to transistor T5 was ‘0’. The transistor had inputs 0, 1 and gave an output ‘0’, indicating that the line D _A_ had been selected to distribute the input from the parity check circuit of the layered decoder circuit. Hence, the other lines D _B_, D _C_, and D _D_ were selected to feed that input for other input combinations to S _A_ and S _B_. The input fed at line D (Y in the truth table) was distributed to any four outputs represented by D _0_, D _1,_ D _2,_ and D _3_. The distribution was based on the select inputs S _0_ (S _A_) and S _1_(S _B_). In Figure 2, the select lines are connected to two inverters at the first stage of the DEMUX. Each inverter created the terms given in equation (2). The inverter drove the value of S _0_, and if it was a ‘0’, then the output could be a ‘1’, similar to the S _1_ input. The following transistors drove the input to the outputs based on the bit pattern of S _1_S _0_.

### Bit update circuit

The bit update circuit is an integral part of many circuits, where temporary storage and stored data updates are required periodically. These circuits have memories that will store some predetermined subset of codeword bits, though only one at a time. The circuit uses basic logic gates: the EXOR gate, a latch, and a multiplexer and inverter. It is like a loop operation, where input data bits received are fed into the multiplexer, and it is compared with the previously stored data from the latch. The EXOR gate will help identify new data and is given to the MUX, where the select inputs will ensure the new data is stored in the latch. This recently stored data is then sent to the next section of a large application circuit.

In the proposed circuit, the data input was from the DEMUX circuit, transmitting data bits received. The bit update circuit ensured that new data received was always updated and stored and then distributed through the reverse router to the parallel adder blocks in the decoder through the data bus. The bit update circuit usually works in tandem with two memories, one as an accumulator for a new data set and the other supplies the last iteration’s data. ^18^ These two memories act in an alternating manner. A multiplexer worked like a cross switch to facilitate their alternating operation.

**Figure 3.**
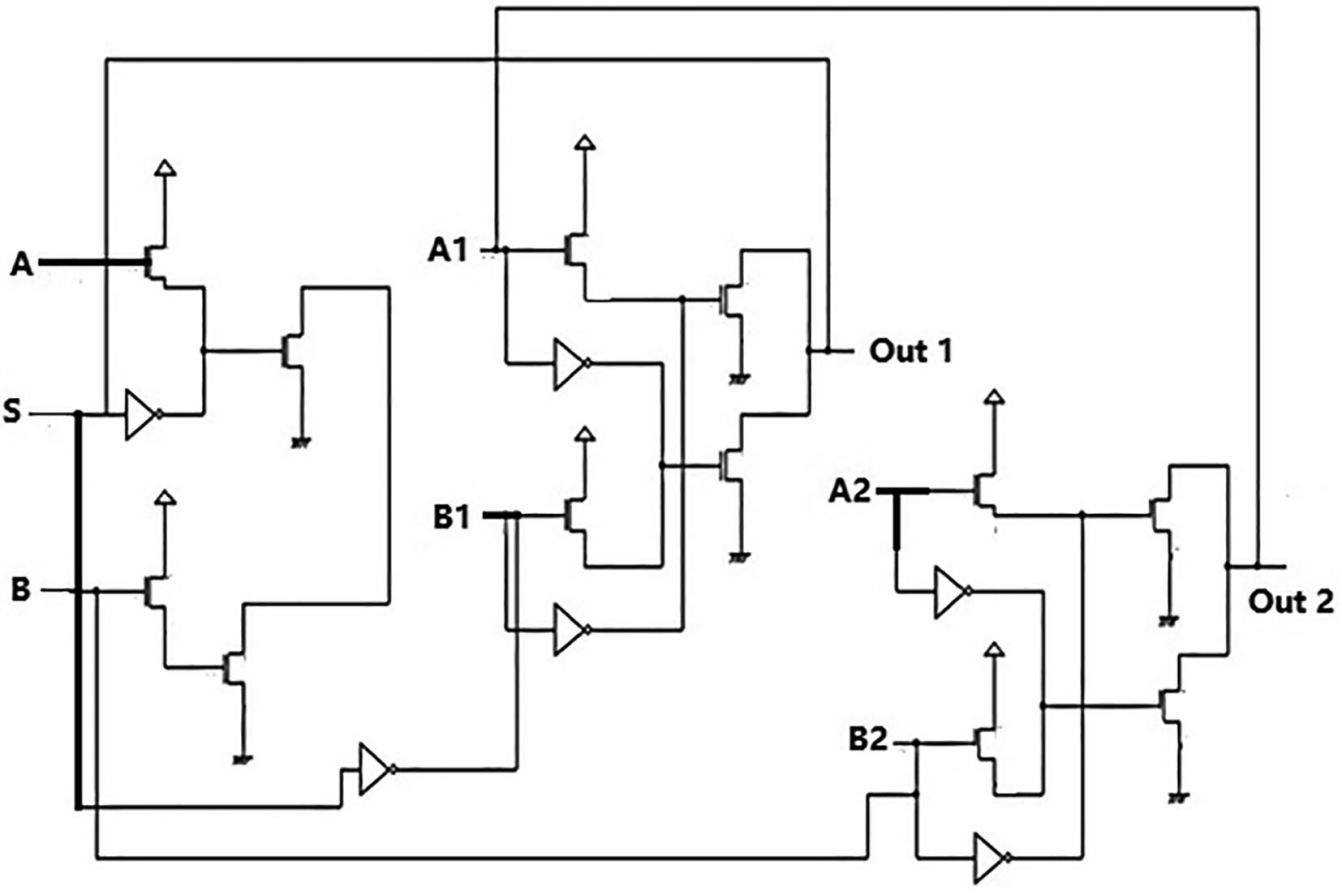
Proposed bit update circuit.

The proposed bit update circuit was designed using the pass transistor logic to reduce the number of transistors. The delay needed to be reduced in the circuit, and hence the technology used was adequate. The circuit shown in Figure 3 comprises a 2 × 1 multiplexer circuit with a latch. The latch acted as the temporary storage or memory for the data bits. The data bit stored in the latch was given to an EXOR gate connected to an AND gate delay circuit. This was to create a delay so that the bits reached the multiplexer within the clock pulse. The EXOR input was also fed to MUX as one of the select inputs.

### The proposed LDPC decoder circuit

**Figure 4.**
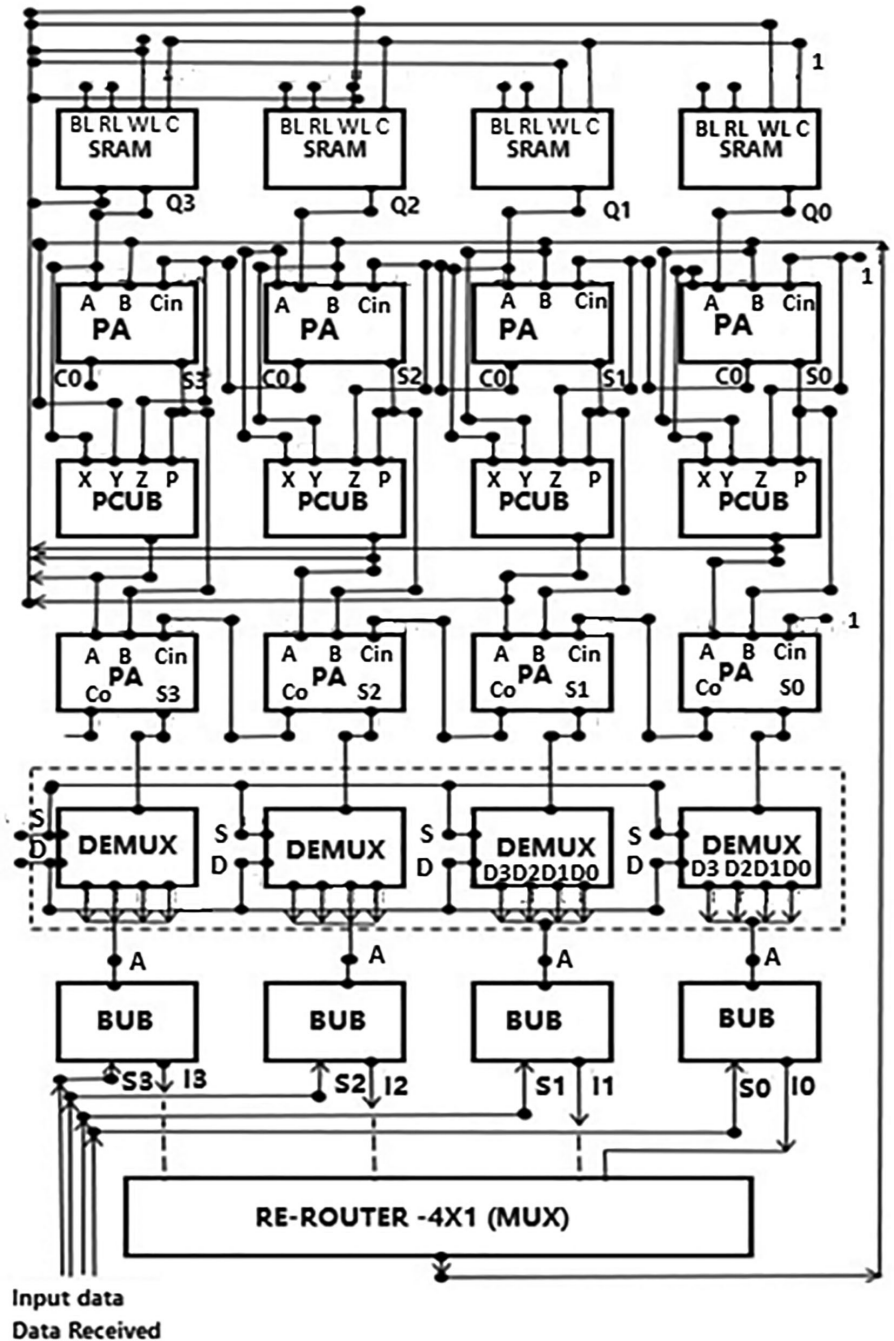
Proposed LDPC decoder architecture.

A proposed decoder architecture is described in this section, which follows the layers of component decoding. The top-level architecture is shown in Figure 4. One type of decoding technique is the layers of components decoding. It generally includes layer-by-layer processing rows of a parity check matrix. ^16^ Each layer is processed sequentially, and the processing of each layer depends on data processed in an immediate previous layer. Decoders using the layered technique are designed to have an inbuilt latency for processing the data between layers. By way of explanation, say if a layer in the parity check matrix needs to be processed, data processed by a previous layer need to be received initially. But it may be that these data are unavailable yet because they are still processed in the previous layer or the data bus and have yet to reach their destination. Latency such as this has an impact on the performance of the decoder. Some problems like this need to be addressed in layered decoding methods. In the proposed circuit, improvements were made to a layered component decoding approach. The method proposed used a plurality of parallel computation blocks coupled to the memory, multiple parity check blocks connected to the computation blocks, and multiple bit update blocks connected to the parity check block. Each bit update block had a memory. The received codeword split in this system, and at least one column/row was grouped and processed.

A low-density parity-check code suitable for efficient hardware implementation was designed with a belief propagation decoder circuit. Codes were arranged according to a sample H matrix whose rows and columns represented the parity check matrix. The decoder circuit had a parity check value that estimated memory, which could be arranged in groups and was logically connected to different data lengths and depths. A parallel adder generated approximate values that were fed to the parity check circuit. The new bitstream generated new values of estimates. These values generated were then stored back in the memory and fed to the bit update circuit. The bit update circuit then updated the new value for the subsequent input data received. Here, layered components decoding was performed by applying the decoding algorithm to each successive layer. Since no particular algorithm was developed, we used a standard to show how the improved decoder works. Applying a decoding algorithm for a particular layer included the use of calculations done in previous layers. The decoding was done using parallelized decoding hardware, and hence its performance may be better than the conventional approach.

The memory block was a local RAM for storing the estimates that were derived within the iteration. These estimates were stored in the memory to save the chip area. The storage memory had one output coupled to one input of the parallel adder. This was connected to the negative input of the parallel adder to provide a subtrahend for subtraction that took place in the parallel adder. The output of the parallel adder was applied to the parity check update circuitry. This block performed the updating of estimates obtained from memory for each of the parity check nodes. The output of the parity check circuit was applied back to the memory to store updated values. It was also applied to the router circuit to update the input nodes’ Log-Likelihood Ratio (LLR). The router circuitry collected multiplexers and demultiplexers that forward the appropriate estimate terms from memory to the corresponding bit update circuit. The bit update circuits were accumulators through which the current values of LLR of the input nodes were maintained from one iteration to the next iteration.

### LDPC operation

The LDPC code was within a parity check matrix H of m × j values and showed a value, H. c = 0 when multiplied by the vector c, considering the Galois field, where c was the transmitted word vector. The Galois field is a finite field that contains a finite number of elements. For each row m of the parity check matrix H, the parity check could be done as H _1_c _1_+H _2_c _2_+ … H _j_c _j_ = 0 over the Galois field. Hence, a parity check equation, the EXOR function of ‘c’, could be written considering the rows of the H matrix having a ‘1’ in their columns.

Referring to Figure 4, soft data received was routed into the decoder system through the data bus. The received data was first routed into the bit update block. Here the data was initialized into its components of a vector. Let us assume the vector for the received data as ‘L’. We defined a set where all the bit columns for a row ‘m’ and the bits in the H matrix have a one in row ‘m’. This makes the checksum for a row over a finite field. The LDPC decoder helps detect errors in the received data when checked for every row in the matrix. When data is received, the values may not be precisely binary values of 1 or 0 but some fractional value and represented by several bits. Hence a probability of whether the bits are 1 or 0 can be represented using the LLR given by:

$$L(r_{j})=\text{log}\left[\begin{array}{c} P(c_{j}=0)\\ P(c_{j}=1) \end{array}\right].$$ (3)

where r _j_ is the input bit value.

As every input bit arrives, the estimated value is written based on the LLR. Initially, an estimate was assumed for the LLR based on the type of channel being used.

A vector ‘R _mj_’ was stored in the SRAM. These were estimates stored in the SRAM after every iteration or cycle of the decoding process and the updated value in the next iteration. The memory stores a few corresponding rows of values of R _mj_, representing vector R values for m rows and j columns from a parity check matrix. For every row, the vector L was written as for the checksum:

$$L({}_{q}mj)=L_{qj}\;‒\;R_{mj}.$$ (4)

The vector was then stored in the BUB. The data were fed into the reverse router block by data buses, where the data was rearranged as required by the system from the BUB. The values of the vector L were given as input to the parallel adder (PA). The other input to the parallel adder came from the memory with the values of the data stored in the form of the components of vector R. The parallel adder performed the operation approximations and subtraction of vector R from L. The results of this subtraction operation in the form of output ‘sum’ were given as input to the parity check circuit and the second set of parallel adders (PA2). A checksum, a sequence of numbers and letters used to detect errors introduced during data transmission, was carried out in the parity check block. The results of this operation were then fed to the second set of parallel adder blocks and the memory block for storage. In the PA2, the computation of the earlier subtraction (R) results and the checksum were added to regenerate the vector L. The new values of L were now sent to the router block to be rearranged into components of vector L. These values were given to the BUB to be stored for the next iteration.

## Results and discussion

The DEMUX and MUX circuits developed here were tested as part of the decoder circuit. The results obtained after simulations at different voltage values and using 180 nm technology are highlighted below, with improvements obtained.

### Demultiplexer (DEMUX)

The 1 × 4 demultiplexers for the LDPC decoder were constructed to have one input D and four outputs D _0_, D _1_, D _2,_ and D _3_. The demultiplexer had two select inputs S _0_ and S _1_. The select inputs formed the decision-maker to connect the input to a selected output. The selection was based on the four possible combinations of the select input, namely, S _0_ = 0 and S _1_ = 1, S _0_ = 0 and S _1_ = 1, S _0_ = 1 and S _1_ = 0, and finally, S _0_ = 1 and S _1_ = 1, representing the binary form 00, 01, 10, and 11. The proposed demultiplexer was simulated to check its characteristics using the Mentor graphics PADS VX.2.7 x86, a CAD tool for 180 nm technology (Open-access software that can perform an equivalent function is DSCH version 2.7for schematic design and MICROWIND version 2.0 for layout analysis).

The string of data bits was given as input D with the select inputs S _0_, S _1_ varied for the four possible combinations. The output satisfied the truth table of the demultiplexer of Table 2. It should also be noted that the voltage rises and falls in Figures 5(a) to 5(c), which are not exactly zero or one. There was a distortion of the signal, but it showed a considerable voltage level to be read as 0 or 1. The voltage variation of 1V, 1.3V, and 1.5V did not significantly affect the output waveforms with only a slight variation in the peak voltage values.

**Figure 5(a).**
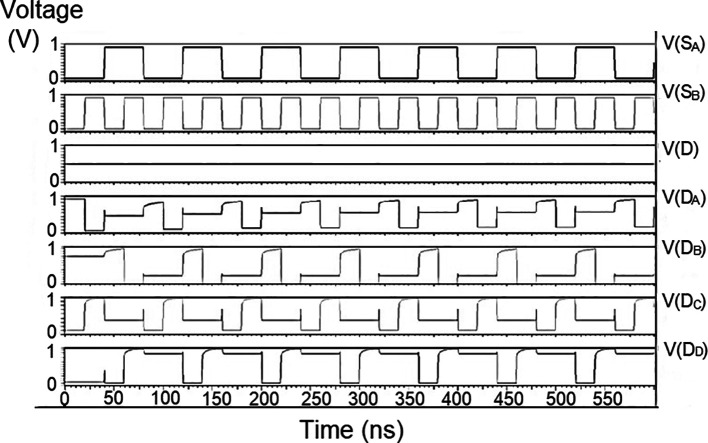
Voltage vs. time simulation at 1V for 180 nm.

**Figure 5(b).**
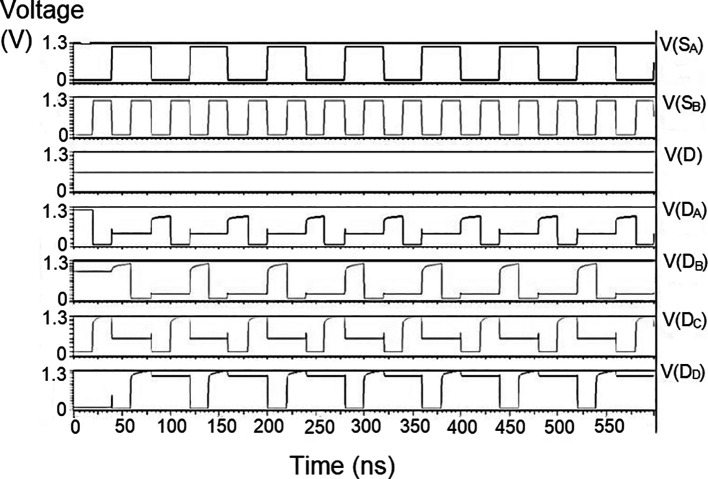
Voltage vs. time simulation at 1.3 V for 180 nm.

**Figure 5(c).**
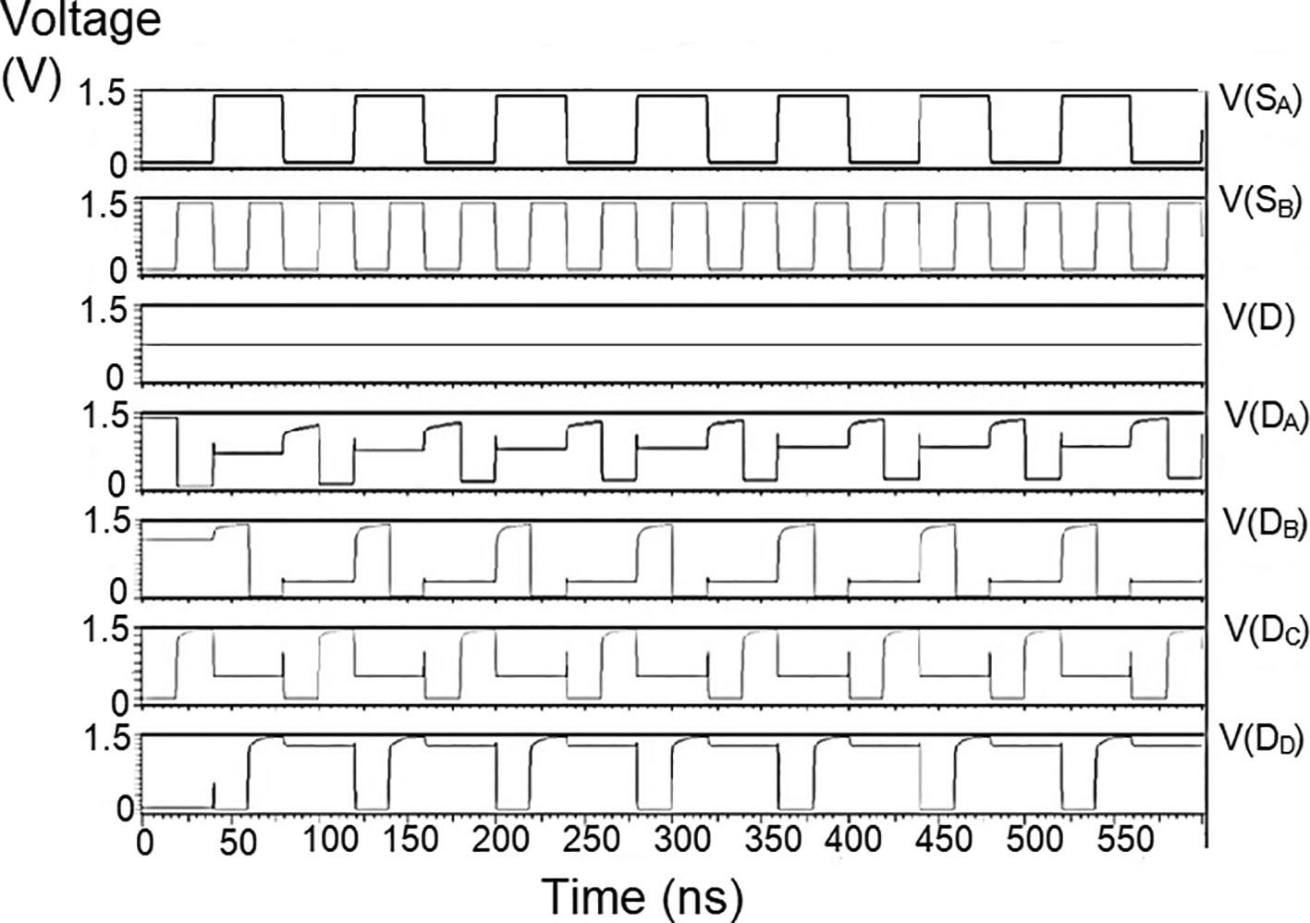
Voltage vs. time simulation at 1.5 V for 180 nm.

The waveforms shown in Figures 5(a) to 5(c) represent the distribution of bits received from the adder circuit (refer to Figure 4). The data’s choice is based on S _0_ (S _A_) and S _1_ (S _B_). The waveforms of D _0_, D _1_, D _2,_ and D _3_ also show the effect of the gates’ switching characteristics, and the peak voltage drops, which is slightly due to the capacitive effect at the input nodes. As the output voltage increases in time, the biasing voltages decrease. A decreasing value of the gate-source voltage reduces the charge density and reduces the output voltage, which does not reach V _DD_.

The output voltage was seen to have a delay in reaching the final voltage. This was due to the parasitic capacitance, the gate channel capacitance between the gate-source and gate-drain terminals. Any switching action in the device leads to the formation of parasitic capacitance. A sudden change of voltage from zero to a high value creates a capacitive effect which can be realized as an RC circuit. Resistance is created, and the device consumes more power to drive the circuit, which depicts a delay in the device’s output voltage. It creates a delay when it drives zero loads. The parasitic delay grows linearly with the number of inputs. This effect was seen in the waveforms for the demultiplexer, which displayed a slow increasing ramp voltage. According to simulation result, the demultiplexer area is 10.5 × 25.555 μm ^2^.

### Multiplexer (MUX)

The reverse router had a multiplexer to transmit data bits from the bit update circuit to the parallel adder through the data bus. The characteristic of the multiplexer was to choose a particular input to be connected to the output. The selection of the input was based on the two select signals. In Figure 3, the schematic of the multiplexer is shown. The multiplexer had four inputs I _A_, I _B_, I _C,_ and I _D,_ and a single output Z. The select inputs were S _A_ and S _B_. Hence the multiplexer was a 4 × 1 MUX. Since there are only two select lines, the possible input lines were four, and the possible combination was S _B_S _A_ = 00, S _B_S _A_ = 01, S _B_S _A_ = 10, and S _B_S _A_ = 11. In line with the truth table in Table 1, if S _B_ = 0 and S _A_ = 0, the line selected would be I _A_ since the combination 00 is for I _0_(I _A_) line. The schematic in Figure 3 is simulated using the test bench. The 180 nm technology was used for the simulation and voltage values of 1 V, 1.3 V, 1.5 V, and 2.5 V. The output observed satisfied the truth table, Table 1. Here the threshold voltage loss restricts the output voltage to the range [0V, V _DD_ – V _Tn_].

The proposed multiplexer circuit was simulated for voltage versus time using 180 nm for input voltages of 1 V, 1.3 V, and 1.5 V, and the output waveforms are shown in Figures 6(a) to 6(c), respectively. Figures 6(a) to 6(c) show the output voltage of the selected input to be given to the output. Even though the output waveform represented the correct selected input, it delayed reaching the maximum voltage. For some inputs, it did not reach the minimum zero value. It was the delay caused by the inverter, and the threshold voltage loss restricted the maximum voltage. Charging the output for a logic one voltage was very slow compared to the transition to a logic 0. The parasitic capacitance increased the charging time from low to high since it was diverted from the output node. The charging of the output capacitance was time-dependent and began as linear as (t/2τ _n_) and then levelled out.

**Figure 6(a).**
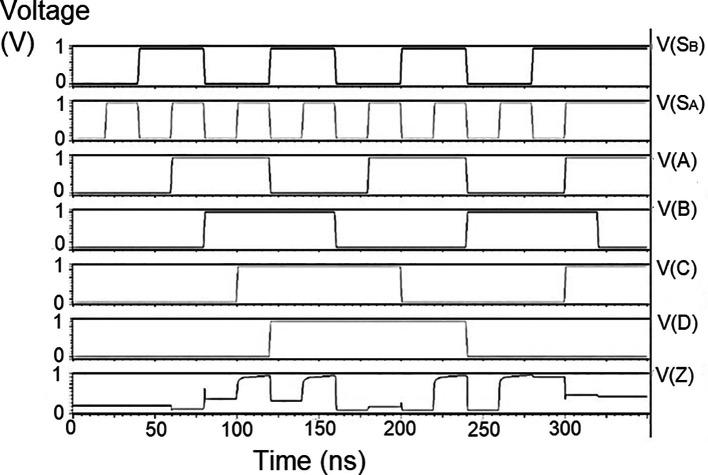
Voltage vs. time simulation at 1V for 180 nm.

**Figure 6(b).**
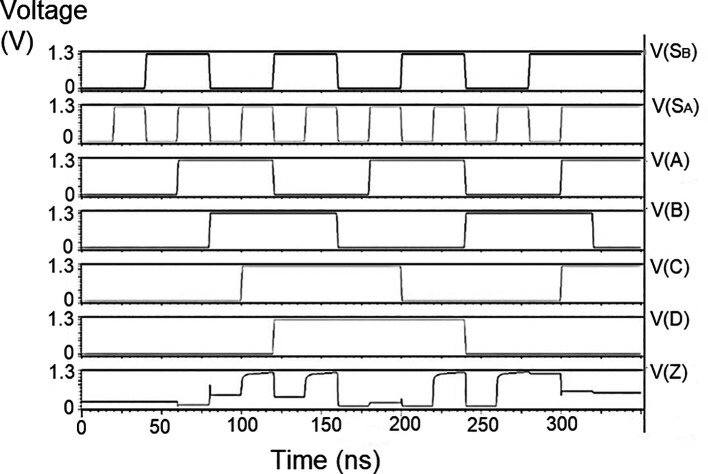
Voltage vs. time simulation at 1.3 V for 180 nm.

**Figure 6(c).**
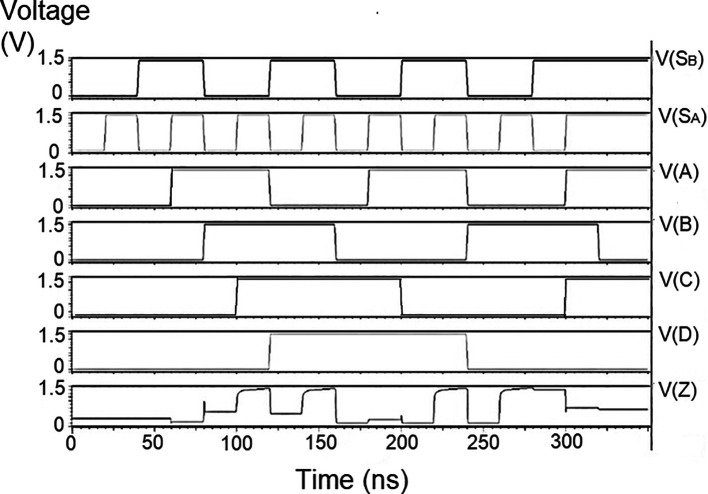
Voltage vs. time simulation at 1.5 V for 180 nm.

Since V _out_(t) increases in time, the device bias voltages V _GS_ – V _DD_ – V _out_ (t) = V _DS_ decreases with time. A decreasing value of V _GS_ reduces the channel charge density, while smaller V _DS_ shows a reduction of the drain-source electric field. This indicates that it is difficult to pass a logic 1 voltage through the n-channel transistor. The spikes seen in the output were caused due to the capacitive coupling of the input to the output by the gate-drain capacitance. As the input suddenly increased from 0 V to V _DD_, the capacitance did not have enough time to drop its voltage instantly. Hence, it would have retained some amount of charge and is seen as spikes of voltage. The proposed multiplexer circuit area is 9.9 × 32.155 μm ^2^.

The multiplexer and demultiplexer circuits were simulated using the SilTerra CEDEC pyxis project of the Mentor graphics CAD tool PADS VX.2.7 x86. The simulation environment was an input voltage value of 1 V, 1.3 V, and 1.5 V for 180 nm technology, tabulated in Table 3. The results showed a low power dissipation in nanowatts. This is because of pass transistor logic, which reduced the number of transistors used and is reflected in the results. A reduced number of transistors (12, 14) led to lower power dissipation and reduced layout area. The delay is only 80 ns and 130 ns for DEMUX and MUX, respectively.

**Table 3.** Results of the simulation for the multiplexer (MUX) and demultiplexer (DEMUX).

Circuit	Input voltage (V)	Power dissipation (nW)	Delay (ns)	Area (μm ^2^)	No. of transistors
Multiplexer	1 V	1.567	80.00	268.32	12
	1.3 V	7.01	80.00	268.32	12
	1.5 V	5.14	80.00	268.32	12
Demultiplexer	1 V	1.537	139.60	318.33	14
	1.3 V	3.660	139.91	318.33	14
	1.5 V	7.067	100.50	318.33	14

Table 4 shows a comparison of the proposed circuit with various published research. It can be seen that the proposed circuit performs better. In terms of power dissipation, the proposed multiplexer circuit has a power dissipation of 7.067 nW, whereas Bousseaud and Negra ^7^ had a value of 5 mW. The approach used by Bousseaud and Negra ^7^ used a transmission gate, while pass transistor logic is used in the proposed circuit. Pass Transistor Logic (PTL) does provide an advantage in the design of circuits with the elimination of redundant transistors. When the number of transistors was reduced, it had a lower power dissipation as each transistor occupied some area and dissipated power. For the DEMUX circuit, the power dissipation produced by Saseendran and Mehra ^6^ had a value of 142 uW, and for the proposed circuit, it was 5.14 nW. The input voltage also tended to be at a lower value of 1.5 V. There was a huge difference in the number of transistors used in the design.

**Table 4.** Comparison of the results with other MUX/DEMUX circuits.

Reference	Circuit	Design	Technology	Area μm ^2^	Power	Supply voltage	Technique	Transistor
Saseendran and Mehra ^6^	DEMUX	1 × 4	90 nm	7482	142 uW	2 V	Adiabatic	36
Bousseaud and Negra ^7^	MUX	4 × 1	65 nm	_	5 mW	1.2 V	Transmission gate	_
Pandey and Akashe ^8^	MUX	2 × 1	90 nm	65.54	1.38 uW	2 V	CPTL	6
Anitha and Jayachitra ^9^	MUX	16 × 1	90 nm	_	5.23 mW	2 V	Transmission gate	162
	DEMUX	1 × 16	90 nm	_	5.23 mW	5 V	Transmission gate	162
Ahn and Kim ^10^	DEMUX	1 × 8	0.35 μm	51200	69.45 mW	3.3 V	RMVL	_
Proposed circuit	MUX	4 × 1	180 nm	318.33	7.067 nW	1.5 V	PTL	14
	DEMUX	1 × 4	180 nm	268.27	5.14 nW	1.5 V	PTL	12

### Bit update circuit

The bit update circuit received new data and then arranged them into its vectors and routes them to the multiplexer as input to the parallel adder. In each iteration of the decoder circuit, the bit update circuit restored new data values after rewriting the data received from the router circuit with data from the transmitter received through the data bus. The bit update circuit was simulated for voltage versus time using 180 nm for input voltages of 1 V, 1.3 V 1.5 V, and 2.5 V, and the output waveforms are shown in the Figures 7(a) to 7(d), respectively.

**Figure 7(a).**
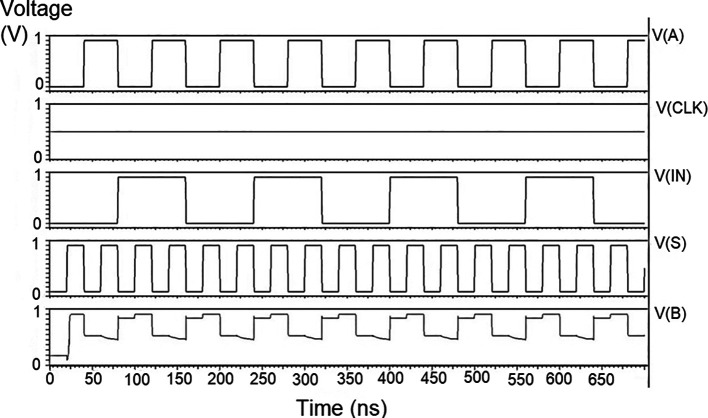
Voltage vs. time simulation at 1 V for 180 nm.

**Figure 7(b).**
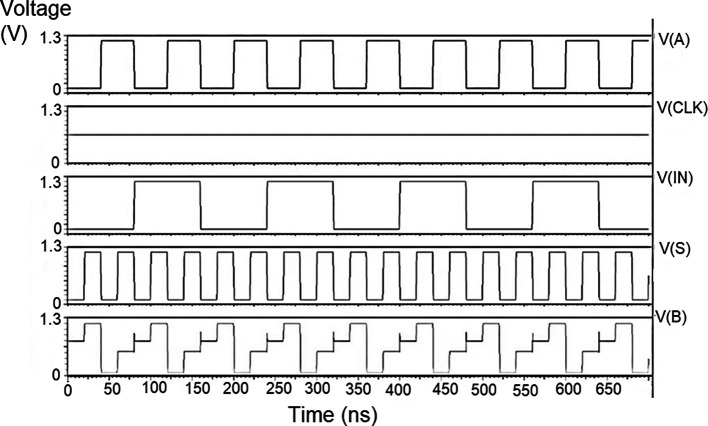
Voltage vs. time simulation at 1.3 V for 180 nm.

**Figure 7(c).**
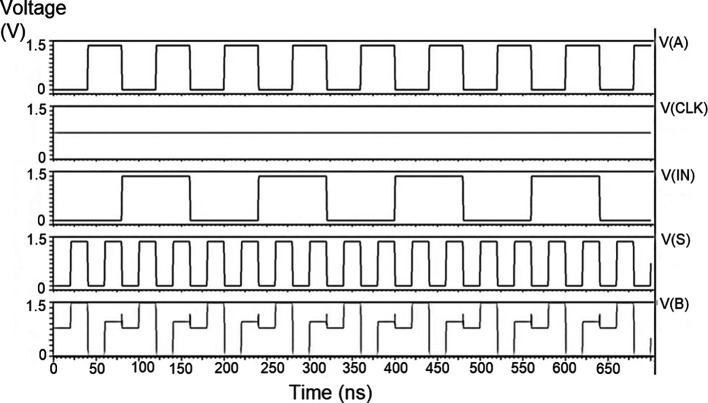
Voltage vs. time simulation at 1.5 V for 180 nm.

**Figure 7(d).**
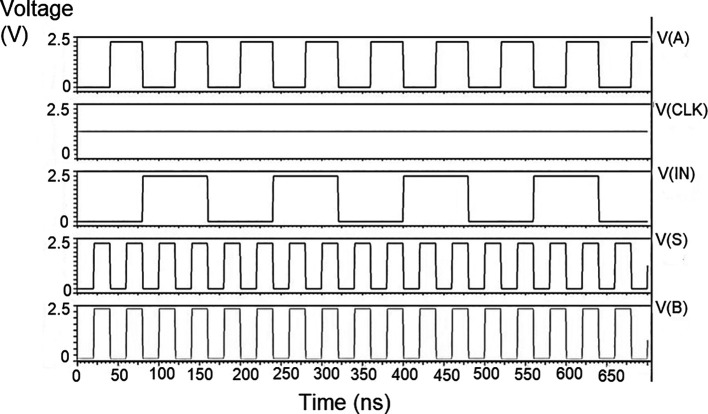
Voltage vs. time simulation at 2.5 V for 180 nm.

Figures 7(a) to 7(d) depict the output obtained for the bit update circuit. The arrival of the clock signal at the input nodes caused clock skew due to the capacitive coupling effect. At the output of 1.5V, it can be observed that the waveform has glitches. Glitches are temporary changes in the value of the output. They were caused due to the skew in the input signals to the gate of the transistor. Glitches can be reduced by gate sizing and path balancing techniques. Propagation of glitches can be reduced by using a smaller number of inverters, which tend to amplify and propagate glitches. At a higher voltage of 2.5 V, the output showed a smooth and expected waveform. The area of the bit update circuit is 46.42 × 14.62 μm ^2^.

### The complete proposed LDPC decoder circuit

The whole LDPC circuit was designed according to Figure 4. The components added to the test bench would be the V _DD_, the ground terminal (GND), DC, and pulse. The DC was the input voltage of 1 V, 1.3 V, and 1.5 V. We needed to set the delay (1ns), initial value (0 V), period (50 ns), the pulse value and the rise time and fall time, and the width of the pulse. The bit pattern to be run through the decoder was also specified. The proposed decoder circuits were simulated for voltage vs. time effect on the output voltage for different input voltages, as shown in Figures 8(a) to 8(c). The input voltages used were 1 V, 1.3 V, and 1.5 V at 180 nm technology.

**Figure 8(a).**
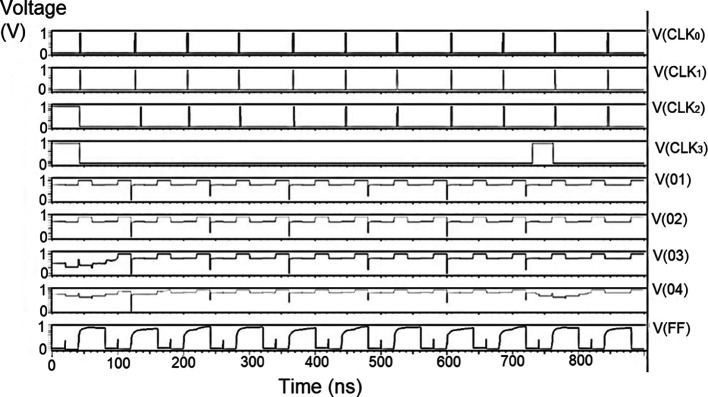
Voltage vs. time simulation at 1 V for 180 nm.

**Figure 8(b).**
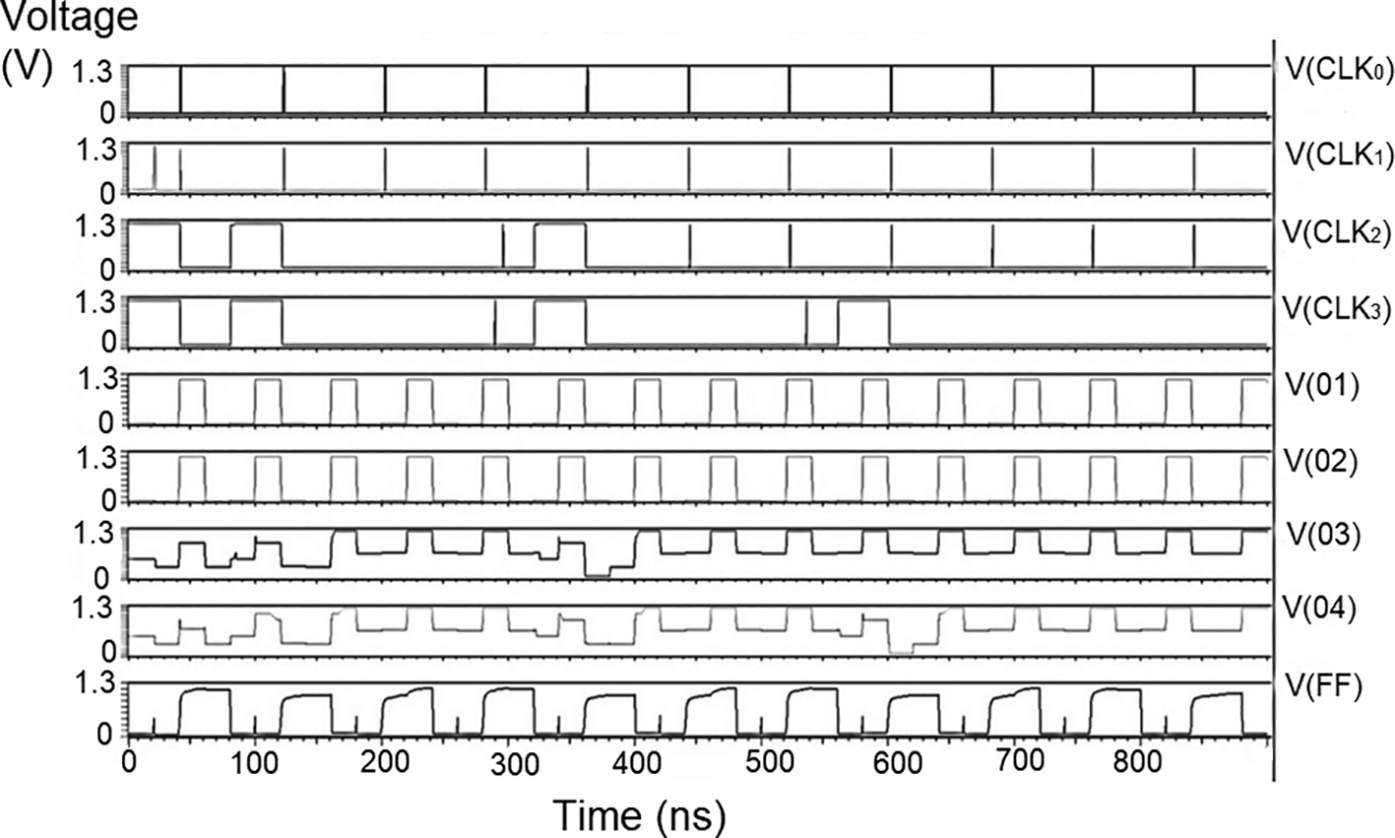
Voltage vs. time simulation at 1.3 V for 180 nm.

**Figure 8(c).**
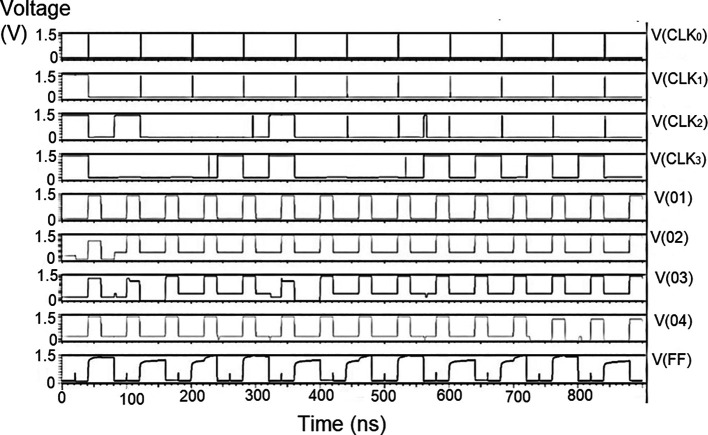
Voltage vs. time simulation at 1.5 V for 180 nm.

The carry inputs to the second set of parallel adders are also shown as check 0 to check 3. The output was measured at various points of the circuit, that is, the output of the memory unit (Vo1), the output of the adder (Vo2), the output of the parity check (Vo3), the output of the router (Vo4), and the final at the reverse router (VoF). It was observed that at the initial points of the check, the output voltage did not suffer from any loss of signal. As the circuit became larger, all effects of power loss come into play due to the different circuits. At the final output (VoF), glitches were observed at regular intervals. This happened to off-pass transistors where the source and drain were initially high and then pulled low. The output of the router circuit shows the waveform reached the peak voltage but did not reach the zero line. This represents the presence of some minimum voltage that did not allow the voltage to reach zero. Practically, the drain current of a CMOS transistor does not reach zero once the voltage of the gate terminal goes below the threshold voltage.

These values are the most updated: the parity check unit block (PUCB) and the values used for the next iteration. ^23^ The flow of data into the circuit with the input of received data at the bit update circuit was tested with bits of data given using the rows from a standard H matrix. Every stage of the movement of the bits through each layer, namely bit update, reverse router through the data bus to parallel adder one and from the adder to the parity check block, a second set of the parallel adders, and the stored data in the memory has been simulated and outputs observed.

### Tabulated results of the proposed LDPC decoder

The results of individual layers and the entire decoder are tabulated in Table 5. Various input voltages were given to observe the effect on the decoder. The decoder circuit designed achieved low power dissipation and a reasonable delay improvement.

**Table 5.** Results of the proposed LDPC decoder circuits (2020).

Circuit	Input voltage (V)	Power dissipation (W)	Delay (ns)	Area (μm ^2^)	No. of transistors
SRAM	1 1.3 1.5	415.286 n 964.608 n 1.424 n	160.00 160.00 160.00	9.5 × 11.69	8
Parallel adder	1 1.3 1.5	61.06 n 295.337 n 762.28 n	60.22 120.00 119.98	7.985 × 20.765	16
Parity checker	1 1.3 1.5	4.476 n 8.774 n 14.811 n	60.025 60.009 60.008	63.200 × 18	46
Demultiplexer	1 1.3 1.5	1.567 n 7.01 n 5.14 n	80.00 80.00 80.00	10.5 × 25.555	12
Bit update circuit	1 1.3 1.5 2.5	635.006 μ 515.219 μ 3.877 n 2.12 n	57.190 79.999 79.999 40.002	46.42 × 14.62	36
Multiplexer	1 1.3 1.5	1.537 n 3.660 n 7.067 n	139.60 139.91 100.50	9.9 × 32.155	14
LDPC decoder circuit	1 1.3 1.5	3.818 n 12.950 n 68.514 n	80.073 80.021 80.023	147.505 × 122.78 = 18110.6639	982

### Comparison of results

In Table 6, the obtained results for the LDPC decoder are compared and analyzed with other published work.

**Table 6.** Comparison of proposed circuit results with published work.

Reference	Technology	Delay (ns)	Power dissipation	Throughput	Frequency	Area
Sipos *et al*. ^19^	90 nm		437.2 mW	7.92 Gbps	-	-
Senthilpari *et al*. ^20^	65 nm	43.2	-	3.9 Gbps	208 M	6.62 mm ^2^
Lee *et al*. ^21^	90 nm	-	517.7 mW	1956.5 Mbps	400 M	5.529 mm ^2^
Bhargava *et al*. ^22^	180 nm	44.53	-	-	22.5 M	-
Proposed circuit	180 nm	80.07 80.02 80.02	3.818 nW 12.95 nW 68.51 nW	12.184 M 12.496 M 12.496 M	-	181 μm ^2^

The proposed circuit performed better in power dissipation than work done by Lee *et al.* ^21^ The power dissipated by the proposed circuit is in nanowatts, while all references are in milliwatts (19, 21). This may be because the proposed circuit was designed using pass transistor logic, which reduced the number of transistors. CMOS circuits dissipate power during switching times.

Hence, reducing the switching activity reduced the power dissipation. Other studies ^19^^–^^21^ achieved 7.92 Gbps, 3.9 Gbps and 1956.5 Mbps for 90 nm, 65 nm, and 90 nm technology, respectively. The proposed circuit simulated for 180 nm obtained the throughput 12.184 Mbps, 12.496 Mbps, and 12.496 Mbps for input voltages of 1V, 1.3V, and 1.5V. The throughput didn’t give a better value because the circuit was designed to function well at lower voltages, which is a trade-off with low supply voltage, lower power dissipation, and smaller area with throughput. We used 1V, 1.3V, and 1.5V, and performance at 1.5V was much better in power dissipation and throughput. At lower voltages, the noise margin becomes critical. The area of the proposed circuit is in nanometres squared, which is also reduced compared to Bhargava *et al*. ^21^ ( Table 6).

## Conclusion

The proposed router circuit, which includes the multiplexer and demultiplexer circuits was designed using pass transistor logic. The proposed circuit gave better power dissipation and throughput performance compared with existing circuits due to the reduced critical path. The circuits were simulated using the Mentor Graphics CAD tool for the design and layout. The results obtained show a significant improvement in power dissipation, area, and delay. For the multiplexer, the improvement in power was 99%, but there was a difference in the technology used. The number of transistors used in the proposed circuit was also significantly reduced, which was the intention of this work. The delay obtained was 80 ns, and the area of 10.5 × 25.55 μm ^2^ for the demultiplexer and 9.9 × 32.15 μm ^2^ was considered small. The designed circuit silicon area utilization ensured reduced delay and power dissipation, making the router circuitry seemingly fitting for use in the decoder circuit. The multiplexer and demultiplexer circuits can be used in an LDPC decoder, which uses the layered approach. The multiplexer received input from the bit update block based on the state of the select inputs. The select inputs chose which data bits needed to be routed to the parallel adder block for the next iteration.

## Data availability

All data underlying the results are available as part of the article and no additional source data are required.
