# Supplementary material for: Low power, less occupying area, and improved speed of a 4-bit router/rerouter circuit for low-density parity-check (LDPC) decoders
Source: F1000Res. 2022 Nov 14;11:7. Originally published 2022 Jan 5. [Version 2] doi: 10.12688/f1000research.73404.2 (PMC9677420; doi:10.12688/f1000research.73404.2)
Supplement: Peer review history

# Peer review: Reviewer response for version 2

**DOI:** 10.5256/f1000research.140044.r155700

**Published:** 2022-11-15

**Authors:** Hamed Farbeh

**Affiliations:** 1 Amirkabir University of Technology, Tehran, Iran

My concerns have been addressed in the revision.

Is the work clearly and accurately presented and does it cite the current literature?

Partly

If applicable, is the statistical analysis and its interpretation appropriate?

Not applicable

Are all the source data underlying the results available to ensure full reproducibility?

No source data required

Is the study design appropriate and is the work technically sound?

Partly

Are the conclusions drawn adequately supported by the results?

Yes

Are sufficient details of methods and analysis provided to allow replication by others?

Yes

Reviewer Expertise:

Reliability, Fault tolerance, Internet-of-Things, Memory technology, Hardware accelerators.

I confirm that I have read this submission and believe that I have an appropriate level of expertise to confirm that it is of an acceptable scientific standard.

# Peer review: Reviewer response for version 1

**DOI:** 10.5256/f1000research.77052.r142900

**Published:** 2022-07-19

**Authors:** Hamed Farbeh

**Affiliations:** 1 Amirkabir University of Technology, Tehran, Iran

The manuscript proposes an efficient circuit for parity check decoder to reduce power consumption, delay, and area of the circuit. The authors tried to optimize the (de)mux circuitry for passing the data bits through the circuit. The evaluation on 180nm technology node show considerable improvement in targeted parameters. The manuscript is fluent and the contributions are clear. My major concerns are as follows:

- The evaluations are performed on outdated 180nm feature size. As we are on less than 5nm technology, how scalable and valid are the observations and improvements of this work? What about the compatibility of the proposal to very smaller technology nodes? A discussion in these directions is necessary.
- The authors reported the absolute value of some parameters, but it does not make any sense when not compared to the state-of-the-art or a reference value. It is recommended to report the comparative values (maybe the percentage of improvements can help).
- By “EXOR” gate, are the authors referring to well-known “XOR” gate?
- Table 4 is strange to me. The authors reported their evaluation on the proposed circuit and the competitors, but they are not evaluated based on the same technology node. When comparing several designs for a circuit, their efficiency is comparable only when evaluated in the same scenario. To my understanding, the authors just used the report of each paper in the table for other schemes and did not implement them in their own experimental platform. If so, the results cannot be technically sound. I strongly recommend the authors to evaluate all schemes in the same evaluation platform.
- The literature review is weak and needs to be more comprehensive. In minimum, the state-of-the-art (DE)MUXs in Table 4 should be discussed. This helps to better highlight the distinction between this work and the existing ones.

Is the work clearly and accurately presented and does it cite the current literature?

Partly

If applicable, is the statistical analysis and its interpretation appropriate?

Not applicable

Are all the source data underlying the results available to ensure full reproducibility?

No source data required

Is the study design appropriate and is the work technically sound?

Partly

Are the conclusions drawn adequately supported by the results?

Yes

Are sufficient details of methods and analysis provided to allow replication by others?

Yes

Reviewer Expertise:

Reliability, Fault tolerance, Internet-of-Things, Memory technology, Hardware accelerators.

I confirm that I have read this submission and believe that I have an appropriate level of expertise to state that I do not consider it to be of an acceptable scientific standard, for reasons outlined above.

# Peer review: Reviewer response for version 1

**DOI:** 10.5256/f1000research.77052.r121437

**Published:** 2022-02-17

**Authors:** Suthendran Kannan

**Affiliations:** 1 Department of Information Technology, Kalasalingam Academy of Research and Education, Krishnankoil, Tamil Nadu, India

I have gone through this manuscript titled “Low power, less occupying area, and improved speed of a 4-bit router/re-router circuit for low-density parity-check (LDPC) decoders’’ which is well written and formatted. Even though some points are against its quality as listed below:

1. Abstract is well written; according to the title, this manuscript occupies less area, which has to be illustrate in the result section of the abstract
2. The introduction is excellent, but if the author adds a text about sections it is well and good
3. Is it necessary to keep tables 1 and 2? If not, remove it
4. Why not give an equation number for the below text equation  “Gate-Source voltage V _GS_ is equal to V _in_, that is:” Page number 4?
5. Figure 5 (a)- 5(c); the output waveform got a skew problem, how to get it and explain in the relevant page?
6. The results and explanations are good, and sufficient materials included.

Is the work clearly and accurately presented and does it cite the current literature?

Yes

If applicable, is the statistical analysis and its interpretation appropriate?

Not applicable

Are all the source data underlying the results available to ensure full reproducibility?

No source data required

Is the study design appropriate and is the work technically sound?

Yes

Are the conclusions drawn adequately supported by the results?

Yes

Are sufficient details of methods and analysis provided to allow replication by others?

Yes

Reviewer Expertise:

Wireless Communication

I confirm that I have read this submission and believe that I have an appropriate level of expertise to confirm that it is of an acceptable scientific standard.
